# Emergency laparoscopic approach without sufficient preoperative decompression for intersigmoid hernia: A case report

**DOI:** 10.1016/j.ijscr.2019.09.036

**Published:** 2019-09-29

**Authors:** Kyota Tatsuta, Shinichiro Miyazaki, Yoshiro Nishiwaki

**Affiliations:** Department of Gastroenterological Surgery, Hamamatsu Medical Center, 328 Tomitsuka, Naka-ku, Shizuoka 432-8580, Japan

**Keywords:** SBO, small bowel obstruction, MDCT, multidetector-row computed tomography, Intersigmoid hernia, Laparoscopy, Emergency surgery

## Abstract

**Introduction:**

In acute care surgery, an increasing number of patients operatively treated for small bowel obstruction undergo laparoscopic procedures. However, intersigmoid hernia is a rare condition. In some reports, surgeons have successfully operated on patients with an intersigmoid hernia via a laparoscopic approach. The laparoscopic approach has the advantage of facilitating simultaneous diagnosis and surgical intervention for intersigmoid hernias. In the laparoscopic approach, sufficient decompression of the small bowel is preoperatively performed in most cases.

**Presentation of case:**

We encountered a patient with an intersigmoid hernia who underwent an emergency laparoscopic approach without sufficient decompression. Because sufficient decompression of the small bowel was not preoperatively performed, it was difficult to establish a working space and visualize the site of obstruction; however, we performed the laparoscopic approach safely, and diagnosis and surgical intervention were possible. Moreover, the postoperative course was uneventful.

**Discussion:**

We successfully performed an emergency surgery using a laparoscopic approach for an intersigmoid hernia without sufficient decompression. The success of the procedure is attributable to the disease-specific surgical strategy, surgical technique, and the recent technological advances in multidetector-row computed tomography.

**Conclusion:**

We believe that careful preoperative diagnosis and strategy can lead to a good outcome and that the significance of emergency laparoscopic approach without sufficient decompression will keep increasing.

## Highlights

• Emergency laparoscopy can be performed without sufficient small bowel decompression.
• MDCT is advantageous for preoperatively recognizing anatomical relationships.
• Careful preoperative diagnosis and emergency laparoscopy can lead to good outcomes.

## 1 Introduction

Intersigmoid hernia is a rare condition of intestinal obstruction due to intestinal incarceration in an abnormal fossa at the attachment of the left leaf of the sigmoid mesocolon to the retroperitoneum. Therefore, a diagnosis of unexplained small bowel obstruction (SBO) used to be conducted previously. Surgery was performed, and in the case of strangulating intestinal obstruction into the intersigmoid fossa, a diagnosis of intersigmoid hernia was established during the operation.

Recently, multidetector-row computed tomography (MDCT) has been attempted for preoperative diagnosis [[1], [2], [3], [4], [5]]. Surgeons have successfully diagnosed intersigmoid hernias preoperatively through advances in MDCT and performed laparoscopic surgeries.

A laparoscopic approach for SBO has many unique challenges such as the introduction of trocars into a distended abdomen and laparoscopic handling of the distended small bowel [6]. To overcome these challenges, sufficient decompression using long intestinal tube insertion was preoperatively attempted. Because of sufficient decompression, surgeons can safely observe whether the small bowel is herniated or not into the intersigmoid fossa. In case of strangulating intestinal obstruction into the intersigmoid fossa, intersigmoid hernias are a definite diagnosis [7]. However, sufficient preoperative decompression is not approved for all cases.

We report the case of a patient with intersigmoid hernia who was preoperatively diagnosed via MDCT and underwent an emergency laparoscopic approach without sufficient decompression. The work has been reported in line with the SCARE criteria [8].

## 2 Presentation of case

**Fig. 1:**
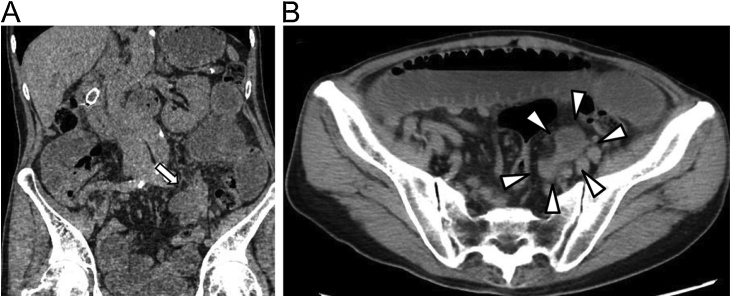
Preoperative multidetector-row computed tomography. (a) The sac-like mass suggests that a closed loop of the small bowel exists in the sigmoid colon dorsum. (b) A cluster of dilated small bowel loops resembling the figure “8.”

An 86-year-old man with constipation for a few days was admitted to our hospital. He had pancreatic cancer; however, he did not expect to undergo surgery or chemotherapy because of old age. His vital signs were normal upon admission. He displayed abdominal distension and tenderness while palpitating the lower abdomen. His laboratory data were almost within normal ranges, except for slightly increased C-reactive protein levels (1.40 mg/dl). A plain abdominal X-ray revealed bowel distention and air-liquid levels. We suspected SBO and performed MDCT. MDCT revealed small bowel dilatation and SBO in the left lower abdomen. Detailed examination of the location of the obstruction revealed that it was located in the sigmoid dorsum and the buckling up curve of the small bowel loop in the edema and that it resembled the figure eight (Fig. 1). We diagnosed the patient with intersigmoid hernia. He continued to display abdominal distension for a few days. Therefore, we performed emergency surgery on the same day.

**Fig. 2:**
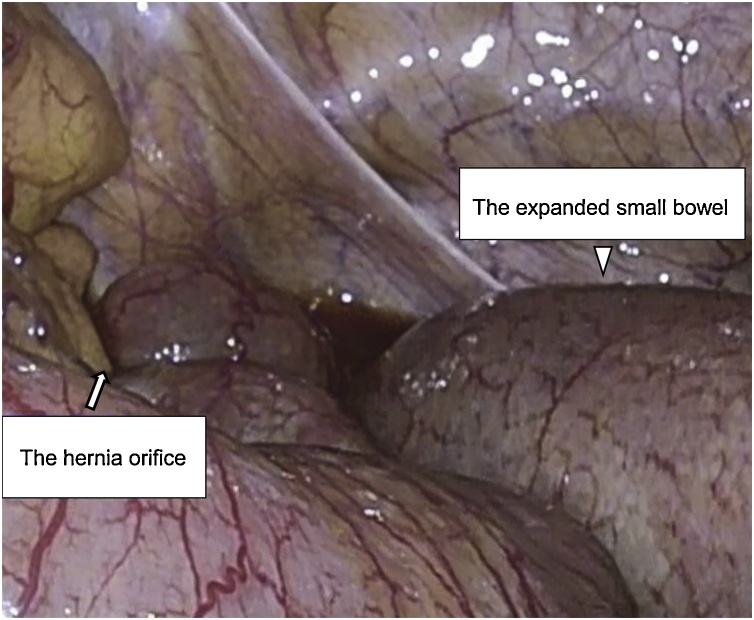
Intraoperative findings.

To conduct laparoscopy, a 12-mm port was inserted via the periumbilical region using the open technique and an intra-abdominal pressure of 8 mmHg was established using carbon dioxide insufflation. Under laparoscopic observation, an additional 5-mm port was inserted via the right lower quadrant and the left abdomen. No dissemination nodule or metastatic lesion was observed in the abdominal cavity. We followed the expanded small bowel and observed that it extended to the left lower quadrant. Because of the dramatic small intestinal expansion, we inserted an additional 5-mm port via the right abdomen. The expanded small bowel was herniated into the intersigmoid fossa, and there was no adhesion or fluid collection (Fig. 2). Following a preoperative diagnosis using MDCT, a definitive diagnosis of intersigmoid hernias was established. We withdrew the expanded small bowel to protect it and opened the intersigmoid fossa. The hernia orifice was large, and there was no possibility of SBO to increase; therefore, it was acceptable to leave the hernia orifice open. The total operation time was 86 min, and the postoperative course was uneventful. The patient has not experienced recurrence for half a year since the surgery.

## 3 Discussion

Two important clinical issues can be noted in this case study. First, the emergency laparoscopic approach for intersigmoid hernia can be performed if sufficient decompression of the small bowel is not preoperatively achieved. Second, this study notes the utility of preoperatively recognizing anatomical relationships using MDCT.

In our case, we successfully performed the emergency laparoscopic approach without sufficient decompression. This success was because of the disease-specific characteristic of intersigmoid hernia and surgical strategy and technique.

Benson classified herniation of the sigmoid mesocolon into three types: (1) intersigmoid hernias, (2) transmesosigmoid hernias, and (3) intramesosigmoid hernias [9]. Based on this classification, our patient was diagnosed with intersigmoid hernia. Generally, intersigmoid hernias relatively have the hernia fossa, and the small bowel within the fossa is not long and is unlikely to be intestinal ischemia.

Because of the disease-specific characteristic, previous reports have demonstrated the utility of the laparoscopic approach for diagnosing intersigmoid hernias. We identified relevant studies by searching the PubMed database using the terms “intersigmoid hernias.” We examined 11 reports but excluded reports that were only abstracts. We also searched the Japan Medical Abstracts Society using the terms “intersigmoid hernias” and examined seven reports but excluded reports that were only abstracts. Information was extracted from the 18 eligible reports (31 cases including our case; Table 1) [7,[10], [11], [12], [13], [14]]. In 15 of these cases, the surgeons safely performed the laparoscopic approach, and diagnosis and surgical intervention were possible. Among these cases of emergency surgeries, four cases, including the present case, underwent the emergency laparoscopic approach without sufficient decompression. Although there was difficulty in establishing a working space and visualizing the obstruction site, the approach was safely performed, resulting in diagnosis and subsequent surgical intervention. The postoperative course was uneventful in all four cases.

**Table 1:** Case reports of Intersigmoid hernias.

Sex	
Male	22
Female	9
Age (years)	56.3 (26–96)
Surgical procedure	
Laparotomy	14
Laparoscopy	15
Laparoscopic-assisted	2
Bowel resection rate	9.7% (3/31)
Treatment strategy	
Emergency	11
Laparotomy	4
**Laparoscopy**	**7**
Cases that were shifted to surgery after conservation treatment	20
Laparotomy cases	10
Laparoscopy cases	8
Laparoscopic-assisted cases	2
Emergency approach	
Sufficient decompression of the small bowel preoperatively	
Laparotomy cases	0
Laparoscopy cases	2
Insufficient preoperative decompression of the small bowel	
Laparotomy cases	4
**Laparoscopy cases**	**4 (our case)**
Cases that were shifted to surgery after conservation treatment	All cases

These four cases share an important surgical strategy. It is important to preoperatively calibrate the lumen of the expanded small bowel. Zermatten et al. proposed that the probability of having to switch to laparotomy increases if the intestine is dilated by more than 4 cm [15]. In our case of emergency laparoscopic approach without sufficient decompression, the intestine was dilated up to 2.8 cm, and we safely performed the laparoscopic approach by employing postural changes. If the intestine is dilated by more than 4 cm, we believe that it is critical to attempt the laparoscopic approach without sufficient decompression. Such measures may include opting for laparotomy in high-risk patients or being prepared to convert to laparotomy early.

Using an appropriate surgical technique resulted in performing the emergency laparoscopic approach without sufficient decompression successfully. Surgeons must not grasp the small bowel but instead grasp the mesentery while establishing a working space and then withdraw the expanded small bowel to protect it. In 1991, Bastug et al. reported the first case of SBO treated using laparoscopy [16]. Despite this early report, laparoscopic approach for SBO did not become widespread because the risk of injury to the distended and thin-walled bowel [17]. Furthermore, the laparoscopic approach for SBO is associated with a higher risk of bowel injury, and surgeons should use strategies to mitigate this risk [18]. Therefore, we believe that for employing our recommended surgical technique, it is necessary to safely perform the emergency laparoscopic approach without sufficient decompression.

Preoperative diagnosis of intersigmoid hernia is difficult because CT only shows wall thickening, edema, or tapered narrowing of the small bowel at the left dorsal to the sigmoid colon. Recent technological advances of MDCT allow for the additional standard multiplanar reformation of the views to the coronal and sagittal planes and improve visualization of anatomical structures, thereby contributing to greater diagnostic accuracy. Takeshita et al. reported a patient with intramesosigmoid hernia who was diagnosed using MDCT preoperatively [19]. They reported three characteristic findings: (1) a sac-like mass, suggesting that a closed loop of the small bowel existed in the sigmoid colon dorsum, (2) a cluster of dilated small bowel loops resembling the figure eight, and (3) dilated and stretched mesenteric vessels and mesenteric fat exhibiting a radial distribution converging toward the medial side. Liao et al. also emphasized the ability of MDCT to preoperatively diagnose herniation involving the sigmoid mesocolon [20]. Although our case was preoperatively diagnosable, we did not recognize all three characteristic findings. However, once we investigated the preoperative images of MDCT in detail postoperatively, our case corresponded to all the characteristic findings. Therefore, using MDCT, the surgeon can proceed with the laparoscopic approach as early as possible, with no priority for the specific diagnosis of the cause of obstruction. Timely surgical intervention minimizes the time-dependent risks of bowel strangulation, ischemia, and infraction.

## 4 Conclusion

The emergency laparoscopic approach for intersigmoid hernias is useful if sufficient decompression of the small bowel is not preoperatively achieved; moreover, recognizing anatomical relationships using MDCT preoperatively is also advantageous. Going forward, as the laparoscopic approach is used more frequently for internal hernias, the adaptation of the laparoscopic approach for acute abdomen conditions will continue to spread.

## Sources of funding

The study sponsors had no such involvement.

## Ethical approval

Investigative involvement and examination were unnecessary in our case, so the approval of the Ethical Review Board was unnecessary.

## Consent

We obtains informed consent of the patient for our study.

## Author contribution

Shinichiro Miyazaki designed the study, and wrote the initial draft of the manuscript. Yoshiro Nishiwaki contributed to analysis and interpretation of data, and assisted in the preparation of the manuscript. All other authors have contributed to data collection and interpretation, and critically reviewed the manuscript. All authors approved the final version of the manuscript, and agree to be accountable for all aspects of the work in ensuring that questions related to the accuracy or integrity of any part of the work are appropriately investigated and resolved.

## Registration of research studies

We enter the name of the registry, and the unique identifying number (UIN) of your study is researchregistry5051.

## Guarantor

Naoto Yamamoto had access to the data, and Naoki Unno controlled the decision to publish.

## Availability of data and material

Data sharing is not applicable to this article as no datasets were generated or analyzed during the current study.

## Provenance and peer review

Not commissioned, externally peer-reviewed

All authors do not have any financial and personal relationships with other people or organisations that could inappropriately influence (bias) their work.

## Declaration of Competing Interest

All authors do not have any financial and personal relationships with other people or organisations that could inappropriately influence (bias) their work.
